# Return of comprehensive tumour genomic profiling results to advanced cancer patients: a qualitative study

**DOI:** 10.1007/s00520-022-07272-3

**Published:** 2022-07-09

**Authors:** Megan C. Best, Nicci Bartley, Christine E. Napier, Alana Fisher, Mandy L. Ballinger, David M. Thomas, David Goldstein, Katherine Tucker, Barbara B. Biesecker, Phyllis Butow

**Affiliations:** 1 Institute for Ethics and Society, University of Notre Dame Australia, Sydney, NSW Australia; 2 Psycho-Oncology Co-Operative Research Group (PoCoG), University of Sydney, Sydney, NSW Australia; 3 Garvan Institute of Medical Research, Sydney, NSW Australia; 4 School of Psychology, University of Sydney, Sydney, NSW Australia; 5 University of New South Wales, St Vincent’s Clinical School, Sydney, NSW Australia; 6 Prince of Wales Hospital, Dept of Medical Oncology, Sydney, NSW Australia; 7 Prince of Wales Medical School, Hereditary Cancer Centre, University of New South Wales, Prince of Wales Hospital, Randwick, NSW Australia; 8 Ethics and Disability Studies, RTI International, Washington, DC USA

**Keywords:** Comprehensive tumour genomic profiling, Cancer, Return of results, Patient perspectives, Psychological responses

## Abstract

**Purpose:**

The introduction of comprehensive tumour genomic profiling (CGP) into clinical oncology allows the identification of molecular therapeutic targets. However, the potential complexity of genomic results and their implications may cause confusion and distress for patients undergoing CGP. We investigated the experience of advanced cancer patients receiving CGP results in a research setting.

**Methods:**

Semi-structured interviews with 37 advanced cancer patients were conducted within two weeks of patients receiving CGP results. Interviewees were purposively sampled based on CGP result, cancer type, age and gender to ensure diversity. Themes were derived from interview transcripts using a framework analysis approach.

**Results:**

We identified six themes: (1) hoping against the odds; (2) managing expectations; (3) understanding is cursory; (4) communication of results is cursory; (5) genomics and incurable cancer; and (6) decisions about treatment.

**Conclusion:**

Despite enthusiasm regarding CGP about the hope it provides for new treatments, participants experienced challenges in understanding results, and acceptance of identified treatments was not automatic. Support is needed for patients undergoing CGP to understand the implications of testing and cope with non-actionable results.

## Introduction

It is widely recognised that cancer is initiated in the genome [1]. Improved understanding of the tumorigenesis process has allowed the development of more effective approaches for reducing cancer morbidity and mortality. Comprehensive tumour genomic profiling (CGP) allows the identification of molecular characteristics in tumour tissue that can be targeted with inhibitor drugs [2]. The use of such agents allows clinicians to treat only patients who will likely benefit, thus avoiding unnecessary side effects in others.

While, in many ways, CGP is similar to common genetic tests undertaken in oncology to identify relevant treatments [3], the range of potential results adds an extra layer of complexity that can confuse and distress patients and their families. Apart from actionable findings to inform treatment decisions, results may include the identification of molecular characteristics with uncertain therapeutic potential, leading to uncertainty for both patient and clinician. Occasionally, CGP identifies germline changes that have implications for biological relatives. Patients may be unaware of this possibility and therefore be unprepared to receive these findings [4]. Disappointment with non-actionable findings, findings of unknown significance or lack of access to trial drugs may be difficult for patients to cope with, particularly if other therapeutic options have been exhausted [5, 6]. Thus, the negative psychosocial impacts of CGP are of concern.

Tumour testing of breast cancer patients has been shown to reduce self-efficacy and increase levels of depression, anxiety and uncertainty. Researchers have suggested that this may be due to the disempowerment of patients regarding confidence to navigate cancer care due to a poor understanding of the process. [7, 8]. Similarly, our group reported that advanced cancer patients who received non-actionable CGP results had increased distress and lower satisfaction with their decision to undergo CGP than patients with actionable results [9]. Those with actionable results who were offered treatment within a research programme reported lower anxiety and depression and higher hope than those offered treatment through their (community) oncologist. Other studies have shown that patients do not regret the decision to undertake CGP, even in the absence of positive results [10].

Pre-test education is not standard for patients undergoing CGP [11], and the need for more research to inform the development of tools to reduce anxiety and negative outcomes has been identified [4, 5]. Thus, the purpose of this study was to qualitatively explore the experience of advanced cancer patients receiving and responding to diverse CGP results, in order to identify the areas where they may require support.

## Methods

The Molecular Screening and Therapeutics (MoST) programme is an Australia-wide cancer genomic study recruiting adult (≥ 18 years) patients with pathologically confirmed advanced or metastatic solid cancers. To be eligible, patients need sufficient accessible tissue for CGP, and to either be receiving the last line of treatment or to have exhausted therapeutic options [12]. The programme uses a customised bioinformatic pipeline to identify potential treatment targets for patients. The American College of Medical Genetics and Genomics (ACMG) guidelines are used when considering what is potentially actionable in the germline arising from tumour testing [12]. Participants are referred by their community oncologist, who receives the results and supervises treatment choices.

MoST participants undergo CGP and can receive one of three results: (1) actionable, with a clinical trial available through MoST (actionable – MoST sub-study); (2) actionable, no clinical trial available through MoST (actionable – other treatment); or (3) no actionable variant (NAV). If there is a relevant therapeutic trial available through the MoST programme for actionable findings, participants are offered enrolment. Notably, germline findings are processed through MoST, but at the time this study was conducted, no participant had received germline results through the programme.

The Psychosocial issues in Genomic Oncology (PiGeOn) Project is a longitudinal, mixed-methods sub-study of the MoST programme which investigates the psychosocial, ethical and behavioural implications for patients undertaking CGP [13]. Participants consent to participate in the PiGeOn Project while consenting to the MoST programme. The consent process involved a verbal explanation of the study by a researcher, accompanied by written information about the study purpose and processes. Both studies were approved by the St Vincent’s Hospital Human Research Ethics committee (Reference HREC/16/SVH/23).

The PiGeOn Project collects questionnaire data from all participants and conducts semi-structured telephone interviews with a subset of participants at baseline, within two weeks of receiving results (8–10 weeks), and 2 months later. This paper reports on interviews from the second time point (post-result-receipt). Interview participants were purposively sampled based on CGP result, cancer type, age and gender to ensure a heterogeneous sample.

Interviews were conducted between November 2017 and May 2019 by two researchers (NB, AF), continuing until data saturation (no new information after three consecutive interviews) [14]. Interviews explored participant responses to the return of results and subsequent treatment decisions and lasted an average of 24 min. Questions were developed iteratively to explore themes identified during data analysis. These primarily concerned the reasoning for decisions made regarding treatment. Demographic details were collected by the parent study.

Interviews were audio-recorded, transcribed verbatim and analysed by framework analysis [15]. Individual coding of transcripts was completed by three researchers (MB, PB and NB) to determine a coding tree, which was applied to further transcripts and developed into themes. Rigour was derived from successive discussions and review of the coding process by researchers until theoretical coding was complete. Differences were resolved through discussion, with the multidisciplinary nature of the research team (psychology, medicine) ensuring reflexivity [16].

## Results

Thirty-seven participants were interviewed. Interviewees were, on average, 61.9 years old, and 49% were female. There was a predominance of rare cancer diagnoses, and an actionable result was available for 67.6% of participants – 27.1% with a clinical trial available through MoST and 40.5% with another treatment (see Table 1).

**Table 1:** Participant demographics

Characteristics	Interviewees (*n* = 37) (*n*, %)
**Sex** Female	18 (48.6)
**Married**	58 (75.7)
**Parent**	29 (78.4)
**Education** Secondary school Vocational training University	15 (40.5) 6 (16.2) 16 (43.2)
**Accessibility/remoteness index of Australia**^a^ Major city Inner regional Outer regional	22 (59.5) 8 (21.6 7 (18.9)
**Cancer incidence** Rare (< 6 cases/100,00) Less common (6–12 cases/100,00) Common (> 12 cases/100,000)	28 (75.7) 4 (10.8) 5 (13.5)
**Comprehensive tumor profiling result** Actionable – MoST sub-study Actionable – other treatment No actionable variant (NAV)	10 (27.1) 15 (40.5) 12 (32.4)
**Multiple primary diagnoses**	5 (13.5)
**Previously visited a family cancer clinic**	5 (13.5)
**Previous genetic testing**	6 (16.7)
**Age (years)** Mean (SD) Median (IQR) Range	61.9 (7.7) 63.0 (12.0) 45.0–75.0
**Time since diagnosis (years)** Mean (SD) Range	2.6 (1.6) 0.1–12.4
**ECOG performance status**^b^ Range	0.0–2.0

^a^Derived from self-reported postcode and the Australian Bureau of Statistics Accessibility/Remoteness Index of Australia

^b^ECOG = Eastern Cooperative Oncology Group

This was a cohort of advanced cancer patients who had reached the end of their therapeutic options and were undergoing CGP to see whether a novel treatment could be identified. If not, these patients had a poor prognosis. Despite enthusiasm regarding the hope CGP provided for new treatments, participants experienced challenges in understanding results, and uptake of identified treatments was not automatic. The experience of the cancer trajectory remained dominant. We identified six themes: (1) hoping against the odds; (2) managing expectations; (3) understanding is cursory; (4) communication of results is cursory; (5) genomics and incurable cancer; and (6) decisions about treatment. Illustrative quotes are provided in Table 2 with sub-theme headings, participant study ID and type of result received: (1) actionable, with a clinical trial available through MoST (MoST); (2) actionable, no clinical trial available through MoST but other treatment through oncologist (OT); or (3) no actionable variant (NAV).

**Table 2:** Participant quotes

**Theme**	**Sub-theme**	**Quote**^a^
1. Hoping against the odds	1.1 Hoping against hope	‘In some ways [testing] opens up your life a little bit more, because you don’t have the constant guillotine hanging over your neck.’ (K1320, OT); ‘Hopefully it’s going to save us from having treatments we know just won’t work… and all the side-effects that you would get if you’re taking all that treatment unnecessarily.’ (K256, OT); ‘I was a bit disappointed, but I didn’t really have great expectations because I know that [my cancer is] a nasty one and I know that options are limited.’ (K1129, OT); ‘[CGP] drills down into what the body is up to, which is all good to know.’ (K421, MoST); ‘[My result is] on record, so if anything comes up in the future that’s to do with that particular protein and ovarian cancer, a flag will go up and say, this woman might be suitable for a trial, or something like that. That’s the way I look at it.’ (K1003, OT); ‘Why wouldn’t you continue to do that [check CGP results against new treatments]? If there are changes it might help them in the future, then it makes sense to me to keep checking.’ (K942, OT)
	1.2 Hoping to contribute	‘[My test result] may help others in the future…[and] unless people offer themselves for testing, we wouldn’t have as many results as what we do have.’ (K897, NAV); ‘All this research, I think it’s wonderful and if there’s anything I can do to sort of assist that– I feel quite good about.’ (K1003, OT)
2. Managing expectations	2.1 Need for low expectations	‘Everyone was quite careful [not to raise hopes].’ (K1047, NAV); “It was a bit of a surprise when he told us [my results]. I think he was quite a bit surprised as well.” (K1038, OT); ‘He was keeping it real.’ (K1038, OT)
	2.2 Coping strategies	‘I consciously prepared myself to receive the news that it wouldn’t do anything for me. So that it wouldn’t be a new big blow and it wouldn’t be distressing.’ (K891, NAV)
	2.3 High expectations persisting	‘I am very positive, yes, it wasn’t the result we were hoping for, but, I think I’ve got enough time that there may be—something else will turn up.’ (K897, NAV)
	2.4 No regrets	‘I think you have to leave no stone unturned.’ (K1129, OT)
3. Understanding is cursory	3.1 Confusion regarding how genomics relates to treatment	‘I think I am a bit confused about the implications of what I was told now. Not that it burdens, me, but confused.’ (K281, OT); ‘I didn’t feel confident enough to discuss it with [my family], ‘cause I’m not across all that detail.’ (K421, MoST); ‘I did understand the crux of it, that there was this possibility of an alternative [treatment] pathway. So, in that sense it was confidence-building.’ (K421, MoST)
	3.2 Cursory understanding about germline	‘[Test results give information about] my children and other family members, how [cancer] could affect them. My mother had cancer and if there’s any connection…it could be used to help them as well.’ (K1038, OT)
	3.3 Better understanding	‘I watched [TV show] Australian story last weekend; I’m all up on it now.’ (K281, OT); ‘[wife spent a lot of time at the hospital]…[and] she picked up a huge amount of information.’ (K421, MoST)
4. Communication of results is cursory	4.1 Results not, or poorly, communicated	‘I think [hospital staff are] overworked. It’s very hard to deal with the hospital.’ (K1095, MoST); ‘I knew the results were available a couple of weeks before [I was contacted by the oncologist].’ (K256, OT); ‘I said to him, was it a genetic marker, and he said, oh no, no it wasn’t a genetic marker, it was something to do with a weakness in the cells which meant that they would be able to target a treatment, and that was it, then he said “Look, I’ll organise you to have an appointment”.’ (K274, MoST); ‘They said that I had a mutation in my DNA or something, but it didn’t say it was susceptible to the treatment.’ (K493, MoST); ‘[the CGP report was] barely decipherable.’ (K281, OT);’The patient should own the results.’ (K1095, MoST)
	4.2 Good communication	‘He [the oncologist] described the shape of the cancer cells, and that there is a treatment available for it and then he explained how that treatment works by latching on to the cancer cell and recruiting other cells in the body to fight the cancer.’ (K304, OT); ‘Given the [negative] results, it wasn’t a huge, big discussion.’ (K1047, NAV)
	4.3 Preferred communicator	‘[my oncologist is] across all aspects of [my] treatment.’ (K1047, NAV); ‘I couldn’t get hold of my oncologist, and anyway, I don’t know that he has the experience that the [researchers] have.’ (K1088, MoST); ‘He [my GP] looks at everything about me, rather than just focussing on one thing, so I feel very reassured with him.’ (K421, MoST)
	4.4 Need for more strategies to promote understanding	‘I suggest [the information] needs to be dumbed down.’ (K281, OT)
5. Genomics and incurable cancer	5.1 Cancer is the central issue	‘I was a bit more interested in knowing what my CT scan was showing [than CGP results].’ (K256, OT); ‘I’m more focussed on what’s happening now, not what might be happening in two months’ time.’ (K942, OT); ‘They decided that I wasn’t well enough to take the drugs that were recommended… so we drew a blank there unfortunately.’ (K1189, MoST)
	5.2 Plodding on	‘I don't think about this new treatment. I figure, I’ve been through so many things in the last few years, I have no expectations with anything, I just take each day as it comes, and I’m just dealing with it like that, pretty much.’ (K1038, OT)
	5.3 Genomics is useful	‘The genetic marker proved why the Enzalutamide worked so well.’ (K307, OT)
6. Decisions about treatment	6.1 Clinical decision-making	‘I think most people would decide on the recommendation of their oncologist, wouldn’t they?’ (K942, OT); ‘[Genomic information] probably goes over their head.’ (K307, NAV)
	6.2 Weighing up the decision	[Patient considering issues before deciding whether to proceed with the proffered treatments]. ‘I’ll be interested to know about side effects and cost, and mortality generally.’ (K281, OT); ‘It’s the devil you know is better than the one you don’t.’ (K1088, MoST); ‘I think they’re running out of treatment for my condition, so I’ll try anything.’ (K493, MoST); ‘…they’re pretty significant side-effects, and I’m not keen on another bout of chemotherapy.’ (K304); ‘We are isolated and it’s very difficult to get to [the treatment hospital]. We’ve got two daughters…they can’t be taking time off all the time [to care for me].’ (K488, MoST); ‘Last time I spoke to my oncologist, that treatment was not available for free, and he didn’t say how expensive it was but that’s another bridge I’ll have to cross.’ (K304, OT)

^a^Quotes are identified by participant study ID and type of result received: (1) actionable, with a clinical trial available through MoST (MoST); (2) actionable, no clinical trial available through MoST but other treatment through oncologist (OT); or (3) no actionable variant (NAV)

### Hoping against the odds

Most participants saw CGP as a way of having a back-up when other treatment approaches failed. Testing was a way of having some hope versus no hope, or a way to keep hope alive. Participants hoped for tailored treatments that would be effective and/or have fewer side effects than chemotherapy*.*

Some participants who did not receive an actionable result were aware of having their hopes dashed. This was generally faced with stoicism and acceptance; however, some participants were disappointed on receiving non-actionable results. *‘There was nothing available at this present time to assist me. I was aware that might be the case, but I was hoping against all odds…’ ( K910. NAV).*

Some participants hoped that the testing would increase knowledge about their type of cancer and the tumour make-up, which would lead to more treatment options for them. Participants also hoped for personal benefit from future advances even if no options existed currently or in the short term. This was based on the assumption that researchers would keep checking their results, a service which was not included in this study but for which they were willing to pay, and also that new treatments would appear regularly.

There was a strong sense of altruism in the cohort, with the hope of being able to contribute to future treatments, whether or not actionable results were immediately forthcoming. There was also a prevalent idea amongst the group that they were part of exciting scientific breakthroughs.

### Managing expectations

Researchers and oncologists had emphasised to participants the need for low expectations prior to the receipt of results. They were so successful with this that actionable results were a surprise to some participants. However, participants appreciated this honesty.

Participants also made a point of keeping their expectations low to protect themselves from disappointment, should a non-actionable result be received.

Despite the warnings from their oncologists, some participants persisted in hoping for a cure or had high expectations of an actionable result. In this cohort, the majority of participants had been diagnosed with rare cancer. This seemed to have altered participants’ sense of probability, i.e., that the ‘norm’ did not apply to them, and led to optimistic rather than pessimistic predictions regarding results. Some still hoped for a cure despite negative results.

Regardless of the result outcome, there were no regrets for most participants, as they felt satisfied that they had pursued every possible option for treatment that was open to them.

### Understanding is cursory

Participants were confused about how genomic testing related to cancer treatment, which could mean that they did not discuss their results with others, such as family members and their general practitioner. Despite this poor understanding, a simplistic understanding that CGP would identify targets for possible treatment was enough to encourage them.

Participants also tended to have a limited understanding of what germline changes were, and whether the changes identified were in the blood sample taken for the study and/or the tumour biopsy. This resulted in uncertainty about the implications of their CGP results for family members, primarily that tumour tests may be relevant to blood relatives.

Participants described how their understanding about genomics improved over time during the study, through exposure to media such as television and the study itself. Improved understanding did not change their positive attitude to testing. Often relatives or carers, who had accompanied the participant on the disease trajectory, had a better understanding through the information they had picked up while spending time at the hospital.

### Communication of results is cursory

Many participants described challenges in accessing their CGP results. There were several reasons for this.

Poor coordination between hospital services meant that results could be difficult for participants to access. Complaints were made that the return of results by the oncologist was typically delayed. Even when presenting to the oncologist once results were available, many participants described communication as non-optimal, brief, and uninformative – followed by immediate referral back to the research team. There was an obvious mismatch in these brief encounters between the hope invested in CGP by the patient and the minor interest of the oncologist.

Comprehension of results was often poor, and many participants could not articulate the treatment implications. Within the consultation, miscommunication about germline, immunotherapy and tumour markers appeared to occur – whether this was related to how it was given or received was not clear. Some participants received copies of their CGP report from their oncologist on an ad hoc basis, which was appreciated by those with a sense of ownership of results, despite poor understanding.

In the few cases where communication of results was felt to be good, participants noted that the use of lay terminology and optimistic language from oncologists was appreciated. In some instances, the communication was short and simple, which could be fine if appropriate (for example, with negative results). Participants appeared to have a range of information needs and needed to make sense of their results according to their beliefs about their own tumour.

Some participants preferred their trusted, known oncologist to give them their results, as this person could personalise the information for them. In the case of actionable results, reassurance from the oncologist that pursuing tailored treatment was a good move was appreciated. For non-actionable results, the oncologist could be comforting and able to discuss the next steps for the patient independent of genomics (unlike the researchers). Other participants would have preferred to get their results from researchers in the MoST programme (not possible in this study), on the grounds that their oncologist may be biased against new innovative treatments as they were more familiar with traditional chemotherapy approaches. The researchers were also seen as more ‘expert’ by some participants.

Some participants were more concerned with the character of the healthcare professional who returned results, wanting a holistic, person-centred focus and good people skills, such as in a general practitioner. Others preferred of a combination of these communication styles.

Participants suggested that extra staff were required to help review the information returned to patients and clarify what was communicated in layman’s terms. They would have liked this to include supplementary written information to take home, which was also written in lay terms.

### Genomics and incurable cancer

In the interviews, while the novelty of genomic testing was appreciated, participants kept returning to their cancer diagnosis as the central issue around which all other issues revolved. Immediate issues, such as staging scans, were often of more import than future therapeutic choices.

Some participants expressed a sense of ‘plodding on’ in an uncertain, grim situation, expressing low expectations regarding the testing process, with emotional responses to results muted by emotional exhaustion.

Apart from any therapeutic benefits, participants found genomic testing useful for understanding their own cancer trajectory, for example, previous responses to chemotherapy.

### Decisions about treatment

Participants were asked about how they decided whether to proceed with the novel therapies identified through CGP. In the transcripts, participants described the process by which they worked through the implications of their results, and the information they sought to come to a decision.

After gathering information, some participants made treatment decisions themselves, with others sharing this decision with their oncologist.

While the family were important in the role of advocating for the patient, or being available to discuss results, they tended not to be involved with treatment decision-making. This might be because of privacy reasons or because participants did not want their choices questioned. For example, participants reported that family members were more often concerned than they were themselves about the chance of being randomised to a placebo in a clinical trial. Another reason for excluding family from treatment decisions was their lack of knowledge about genomics and their anticipated lack of comprehension.

Many issues were considered as participants weighed up whether to proceed with the proffered treatments. Despite the strong motivation to find new treatments prompting most of the participants to undergo CGP, uptake of therapy was not necessarily automatic. Other issues such as drug efficacy, travel and accommodation requirements for treatment, carer availability and drug cost were also of interest. These were viewed as barriers to accessing treatment for some. Other participants who were on last-line therapy which was still working, remaining on the known and familiar treatment (‘*the devil you know*’) was the preference. Some participants were just desperate to prolong life now they were at the end of the line, willing to try anything. Others felt there were limits to what treatments they could endure, given their previous experiences. When treatments were available through testing, but the patient was ineligible by the time results were received, patients were very disappointed.

## Discussion

This qualitative study investigated the experience of advanced cancer patients receiving CGP results. We found that, regardless of the test results received, participants were glad that they had joined the study and been tested. The opportunity for CGP was seen as a positive experience, in terms of the hope it gave for extended survival. Even when results were non-actionable, participants were encouraged by their contribution to science and cancer treatment in general, and possibly future treatments for themselves. However, despite this enthusiasm, the decision to access identified treatments was not automatic.

We found that this cohort generally had limited understanding of what CGP involved and the significance of results for treatment or family members. While all participants had given informed consent, receipt of relevant information seemed to vary. There is no way of knowing what study participants are actually told, or how much written content they comprehend. Some patients reported that understanding had improved due to media coverage of genomics, but we note that only 13.5% of patients had previously attended a genetics clinic, and so may have had little previous exposure to genetics. Previous studies suggest that clinicians cannot assume baseline knowledge of genetics and that confusion between somatic and germline testing is not uncommon [17, 18]. While participants reported that self-perceived knowledge improved over time, Adams and colleagues found that objective knowledge of genomics did not improve through contact with a CGP research project alone [7]. These findings suggest that more work is required to identify what is needed to adequately educate patients about their care particularly in the context of informed consent for CGP. Borno and colleagues have suggested that as a minimum both the benefits and risks (including secondary findings and germline changes) should be communicated by clinicians in the context of somatic testing [4]. In this study, management of expectations by both participants and their clinicians ameliorated disappointment. Standardisation of pre-test counselling may avoid unnecessary distress by ensuring that patients understand the likelihood of positive results and the implications of CGP prior to testing [4].

Many participants experienced difficulty in accessing their CGP results. Some of these problems appeared to be a result of poor communication between the research hospital and the community oncologists. There were also problems for some participants in understanding the implications of their results and how they should respond, which has been seen in other cohorts [19]. This may have been compounded by the difficulty some oncologists experience in explaining CGP results, as reported in other studies [20]. This highlights the ongoing need for improved genomic literacy amongst oncologists as mainstreaming genomics becomes more common [21]. Although support will be, to some extent, resource-dependent [22], there may be a role for genetic counsellors in the somatic genomic testing space to facilitate patient understanding of results [4]. Given that over 10% of cancer patients may have germline changes [23] and given the long-term implications of such changes for family members, genetic counsellors would also be able to provide the needed ongoing support for biological relatives. Referral to such services should be considered at the time of CGP result return (if necessary). Enhanced reporting of results, where the genomic result from the laboratory report is presented in language accessible by patients, family members and non-genetic providers, may also be helpful for oncologists in this setting [19].

Despite the desire for access to novel treatments driving the decision to participate [17], decisions regarding whether to act on positive results were made with consideration of a wide range of factors beyond merely the potential for prolonging life. Factors considered included treatment cost, side effects, efficacy and practical considerations such as carer availability and long travelling distances to access treatment. The long-term impact of the cancer trajectory on the lives of participants was notable, and decisions were made within this context.

Concern regarding the impact of precision medicine on cancer patients who receive negative results has been previously expressed [5]. Approximately one-third of our cohort (32.4%) received negative (NAV) results, which is below the average reported in the literature [23]. A psychological assessment of this cohort in a separate study found that the return of negative results was associated with increased distress [9]. A further group of patients who may suffer during this process are those who have positive findings from CGP but are unable to access the identified treatment whether due to ill health or logistics. We identified this experience in this cohort. Currently this group comprises the majority of patients who receive actionable results [24]. It is possible that this group of patients may experience emotional reactions that could influence treatment-related behaviours, such as loss of confidence in ‘non-targeted’ treatments, with the potential to undermine treatment compliance [5, 25] or contribute to a reluctance to undergo somatic testing [4]. We found a high interest in testing and suggest that rather than avoiding CGP in clinical practice, oncologists should consider the support needs of patients who are unable to proceed to new treatments following receipt of CGP results. Oncologist training in returning bad news and involvement of psychologically trained caretakers may help them respond to patient distress associated with the potential lack of treatment options and fear of dying [26].

As a limitation, no participants received germline results during this study, so we were unable to assess the impact of this type of result return. This should be examined in future. This cohort was enrolled in a research study, and participants may have been biased towards novel treatments.

## Conclusion

In this cohort of advanced cancer patients undergoing CGP, attitudes about the testing process were positive overall. Participants receiving actionable results needed to consider the wider implications of treatment before proceeding, and those receiving non-actionable results, or unable to access treatments, while experiencing disappointment, were satisfied that they had explored this option. Patient support during the return of CGP results, in the form of education provision and possible referral to a genetic counsellor, is recommended.

## Data Availability

The data is available from the corresponding author on reasonable request.
